# Data-Driven Learning in High-Resolution Activity Sampling From Patients With Bipolar Depression: Mixed-Methods Study

**DOI:** 10.2196/10122

**Published:** 2018-06-28

**Authors:** Darius Adam Rohani, Nanna Tuxen, Andrea Quemada Lopategui, Lars Vedel Kessing, Jakob Eyvind Bardram

**Affiliations:** ^1^ Embedded Systems Engineering Department of Applied Mathematics and Computer Science Technical University of Denmark Kgs. Lyngby Denmark; ^2^ Copenhagen Center for Health Technology Technical University of Denmark Kgs. Lyngby Denmark; ^3^ Psychiatric Center Copenhagen Rigshospitalet Copenhagen Denmark; ^4^ Faculty of Health and Medical Sciences University of Copenhagen Copenhagen Denmark

**Keywords:** activities, behavior, behavioral activation, bipolar disorder, circadian rhythm, depression, hourly planning, psychotherapy, statistics

## Abstract

**Background:**

Behavioral activation is a pen and paper-based therapy form for treating depression. The patient registers their activity hourly, and together with the therapist, they agree on a plan to change behavior. However, with the limited clinical personnel, and a growing patient population, new methods are needed to advance behavioral activation.

**Objective:**

The objectives of this paper were to (1) automatically identify behavioral patterns through statistical analysis of the paper-based activity diaries, and (2) determine whether it is feasible to move the behavioral activation therapy format to a digital solution.

**Methods:**

We collected activity diaries from seven patients with bipolar depression, covering in total 2,480 hours of self-reported activities. A pleasure score, on a 1-10 rating scale, was reported for each activity. The activities were digitalized into 6 activity categories, and statistical analyses were conducted.

**Results:**

Across all patients, movement-related activities were associated with the highest pleasure score followed by social activities. On an individual level, through a nonparametric Wilcoxon Signed-Rank test, one patient had a statistically significant larger amount of spare time activities when feeling bad (*z*=–2.045, *P*=.041). Through a within-subject analysis of covariance, the patients were found to have a better day than the previous, if that previous day followed their diurnal rhythm (ρ=.265, *P*=.029). Furthermore, a second-order trend indicated that two hours of daily social activity was optimal for the patients (β_2_=–0.08, *t* (63)=–1.22, *P*=.23).

**Conclusions:**

The data-driven statistical approach was able to find patterns within the behavioral traits that could assist the therapist in as well as help design future technologies for behavioral activation.

## Introduction

Unipolar and bipolar depression has a high yearly prevalence in all age groups from children [1], adolescent [2] to older adults [3,4]. The high prevalence imposes significant adverse consequences, including increased mortality, societal costs, loss of productivity, and lower well-being [5,6]. Treatment for bipolar depression consists of pharmacotherapy, psychotherapy, or a combination[7]. One of the most efficient and successful methods of psychotherapy for bipolar depression and many other mental disorders is cognitive behavioral therapy (CBT) due to its short-term consultations and problem-solving technique[8]. CBT may be as effective as pharmacotherapy in unipolar depression [9] and provides long-term protection against relapse. However, it is still unclear whether CBT has that effect in bipolar depression [10]. Further, CBT is complicated and time-consuming, and its effectiveness is dependent on the skills of psychological therapists, who are expensive to train and employ [11]. This leads to long waiting lists for cognitive therapy, resulting in a treatment gap of 56% for depression where less than half of the patients received proper treatment [12].

Behavioral activation (BA) is a more straightforward therapy approach focusing entirely on changing behavior. It can be performed by less trained therapist and junior mental health workers. BA includes activity-monitoring, scheduling and regulation of sleep and daily routines which helps to reduce both depressive and manic symptoms [13,14]. Alone it may be as effective as pharmacotherapy and better than cognitive therapy in unipolar depression [13] but has not been thoroughly tested for bipolar depression [8].

**Figure 1:**
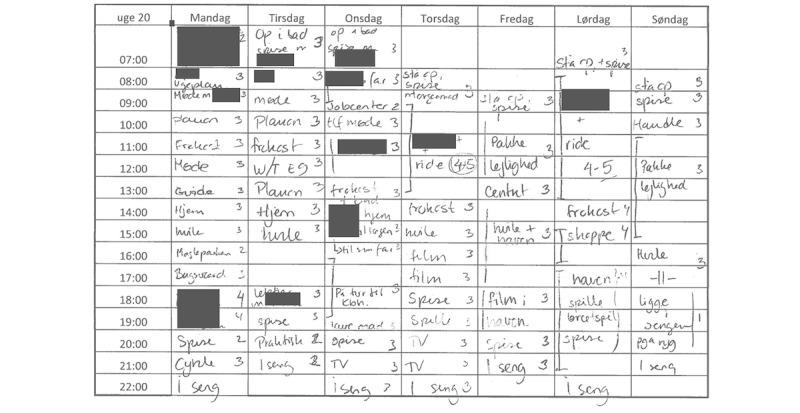
A one-week paper-based behavioral activation schedule, in Danish, from patient P3. Activities have been filled out from Monday till Sunday between 7 am and 10 pm, with a pleasure score from 1-5. Sensitive information has been blurred.

BA relies on the patient to collect detailed hour-by-hour activity information daily. As shown in Figure 1, this is normally done in a paper-based diary detailing activities like “*sleeping*,” “*going for a walk*,” “*meeting with a friend* ” or “*watching TV*.” Each activity is rated with a “pleasure score.” This detailed information is later used by the therapist to identify activities that alleviate, maintain or worsen symptoms and help the patient to change his or her behavior in a healthier direction.

Recent research has suggested using mobile technology to support BA [15-17]. This can have several potential benefits. First, it has been shown that using a mobile app is less subject to retrofitting of self-assessment data as compared to paper-based schedules [18]. Second, recording activities can be done semiautomatic by, eg, using mobile phone-based sensors to collect information on physical activity like walking. Third, by using modern data analysis methods, the system could potentially be able to present recommendations to the patient [19]. As such, mobile technology would have the potential to improve existing BA methods by assisting inexperienced therapists to locate possible healthy reinforcers and to provide the patient with a powerful data-driven insight into their behavior.

However, BA requires the patient to do detailed self-reporting of daily activities. This can be a cumbersome task, whether it is done on paper or a mobile phone. Therefore, to investigate the feasibility of the design of mobile phone-based BA systems, there is a need to understand the details of current BA activity sampling and the kind of insight, which can be gained from such dense activity data.

To investigate this, we present a detailed analysis of a set of paper-based activity schedules filled in by 7 patients with bipolar depression covering in total 2,480 hours of activity sampling. The analysis of realworld BA self-reporting, helps us understand the following relevant factors:

1. What type (categories) of activities are patients doing?
2. What is the relationship between activities and the patient's daily pleasure?
3. What is the relationship between having a good day and the kind of activities done?
4. What is the relationship between circadian rhythm and pleasure?
5. What is the optimal number of different types of activities a patient should do during a day?

Answering these questions generates possibilities of assisting the therapist with data-driven insights and automatically generated suggestions. This could potentially be of great help during consultations and lower the requirements for BA training. Moreover, this kind of insights is equally relevant for the patient him or herself. Finally, even though the use of paper-based schedules does not predict engagement or feasibility of using a mobile phone-based approach, this study provides insight into the details of how patients fill in BA schedules and help the design of technologies for BA.

## Methods

### Participants and Procedure

Paper-based BA schedules were collected from patients diagnosed with bipolar disorder (BD) who experienced a mild to moderate bipolar depression, corresponding to a score on the 17-item Hamilton Depression Rating scale <20, and currently enrolled in BA therapy. The BA therapy was done at the Psychiatric Center Copenhagen, Rigshospitalet, Denmark. Patients were instructed to fill out hourly activities between 7 am and 10 pm on a weekly schema as shown in Figure 1. Patients were asked to fill in the activity details ‘as soon as possible’.

For each activity, the patient registers a *pleasure score* (PS), indicating “*how much did you enjoy doing that activity* ” on a 1-10 numerical rating scale.

A nurse or a psychologist collected schedules. Patients provided informed consent to use their schedules in an anonymized manner for this study. Activity examples from the schedules that are presented throughout the paper are translated from Danish into English for the convenience of the readers. The handwritten activity schedules were transcribed into comma-separated values (CSV) files by categorizing each activity to an *activity category* (AC), with the corresponding PS. Transcription was performed by 2 independent researchers (DAR, JEB). The intercategorization agreement was assessed by Cohen kappa (к).

The ACs consist of 7 distinct classes to cover all types of activities and have been previously presented [15,16]. They were developed in close collaboration with a psychologist by the approach presented in Mørch and Rosenberg and Lejuez et al [20,21] combined with the primary activities introduced in the American Time Use Survey (ATUS) by the Bureau of Labor Statistics [22]. The 7 AC’s are presented in Textbox 1.

### Statistical Analysis

The data analysis was performed in MATLAB version R2017A. From the digitalized CSV files, we investigated the following topics on both individual and group level to gain data-driven insights from the schedules.

#### Activity Categories

The activity categories have not been used in previous analyses of paper-based BA. Therefore, an initial analysis of the 7 ACs was performed by calculating summary statistics including distribution, variability, and their daily average PS.

#### Activity Category on Good Days

To further examine the activity categories we investigated, on an individual level, whether the extent of specific ACs was different between days that had a larger average PS (eg, *good days*) and days with a lower average PS (eg, *worse days*). The grouping into *good days* and *worse days* was done by calculating the median from the patients’ daily average PS. The days above the median were classified as *good days* and vice versa. The amount of AC, weighted by the distance from the median, was then subject to a nonparametric, uncorrected for multiple comparisons, Wilcoxon Signed-Rank for each category. The null hypothesis stated that the number of daily hours of a category in the *worse days* did not differ from the daily hours in the *good days* at a type I error level of alpha=.05.

#### Circadian Rhythm

To keep a circadian rhythm, denoted here as *diurnal rhythm*, have shown a positive statistically significant correlation with mood [23]. This relation is explored by defining *diurnal rhythm* as the most common AC within each hour. The Jaccard similarity coefficient (JAC) was then calculated by matching each hour slot for the day with the corresponding slot in the *diurnal rhythm*. A timeslot match equals a value of one, and the total number of hour slots then divides the summation across all the hours for that day. Hence, a JAC of 1 indicates that the entire day followed the *diurnal rhythm* exactly. JAC of *day*_x_ was correlated with the change in PS (day_x+1_-day_x_). An analysis of covariance (ANCOVA) was then conducted to investigate the within-subject correlation value with JAC as an independent variable, change in PS as the dependent variable and subject ID as a dummy coded grouping variable. The analysis was only done on weekdays since the weekends yield different behavioral characteristics [24].

**A list of the 7 activity categories (AC). The ACs are listed with a brief explanation and with real examples from the patients, in parenthesis.**

Movement: Activities that involve physical activity (eg, “*training*,” “*go for a walk*,” “*horse riding* ”)

Work & education: Task involving work, learning, or treatment (eg, “*work*,” “*lecture*,” “*outpatient consultation* ”)

Spare time: Time spend alone on hobbies or similar (eg, “*relaxing*,” “*watching a movie* ”)

Daily-living: Basic daily activities of living (eg, “*sleeping*,” “*having breakfast*,” “*bathing* ”)

Practical things: Practical daily activities (eg, “*cleaning*,” “*pick up kids*,” “*shopping* ”)

Social: Spending time with others (eg, “*cinema*,” “*concert*,” “*football* ”)

Other: Uncategorized activities (eg, “*transport*,” “*crying*,” “*migraine attack* ”)

#### Activity Category Frequency

Lastly, on a daily basis we sought to investigate whether there was a relationship between the amount of an AC and the resulting average PS. To address this, for each AC we performed a cross-sectional linear regression analysis using the least squares method of the daily hours as a function of the average PS. A quadratic model defined as *dPS_S_=β_0_+β_1_ X_S_+β_2_ X_S_^2^* was fitted the data, where *dPS_S_* represents the daily average PS for patients, β the weightings, and *X* is the number of hours of the specific AC. If the quadratic term was insignificant, we re-ran the analysis as a linear model.

## Results

BA forms from 7 patients were collected, covering in total 24 weeks, 153 days and 2,480 hours of self-reported activity data. Demographics data, level of reporting, and Cohen kappa are listed in Table 1. A total of 4 patients had reported PS.

The transcription of the handwritten activities to the ACs was done with a high agreement across all subjects with mean kappa=.81 among the independent raters. The majority of disagreement was within activities that combined two or more AC’s, such as P6: “*fixing riding equipment* ” which was classified for 1 rater as *Practical things*, and the other *Spare time*. In one case (P7) the value kappa=.66 fell below the threshold of kappa=.75, and therefore subject to a discussion among the raters. The disagreement was related to one recurrent entry labeled “*computer* ” which was falsely classified as *Work & education* by one rater, and afterward was agreed to be *Spare Time* since the computer was used for watching movie series online.

**Figure 2:**
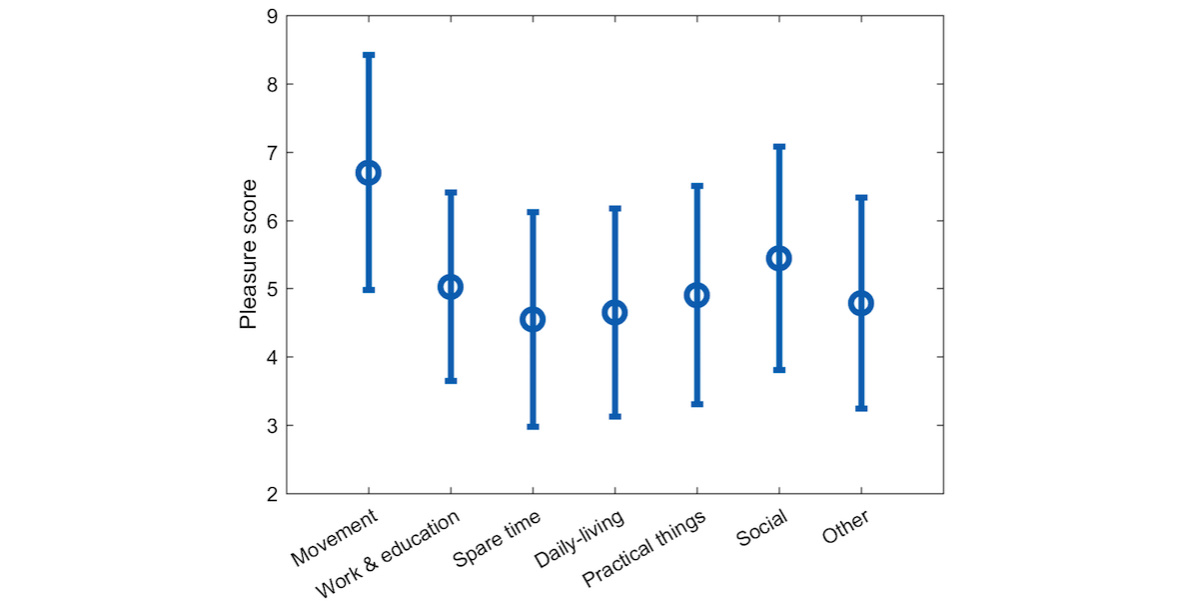
The total average pleasure score is shown for the seven activity categories as a circular marker. ±1 SD is shown as error bars for each average value.

The total average PS for each AC is shown in Figure 2. The distribution in the percentage of the AC for each patient is given in Table 2. The overall AC classifications, in the same order as Table 2 where 6% roughly corresponds to an hour, is 5.11%, 13.48%, 14.70%, 25.36%, 12.20%, 23.97%, and 5.17% respectively. Socially based activities correspond to 534/2230 (24.0%) of all activities, while movement-related activities are on average represented less than 115/2230 (5.1%) which corresponds to under an hour daily. However, *Movement* was on average the activity that scored highest on the PS with a mean of 6.70 (SD 1.72).

**Figure 3:**
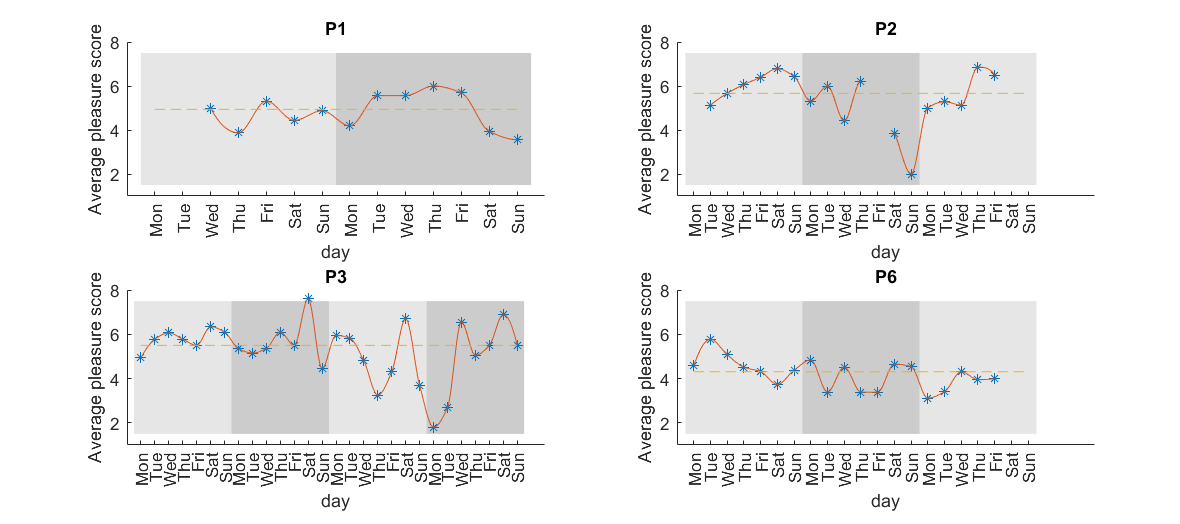
The avg PS for each day, interpolated by a shape-preserving piecewise cubic interpolation, is shown for all valid subjects. The background color indicates change in week. Empty values indicate missing data entry from the patient.

**Figure 4:**
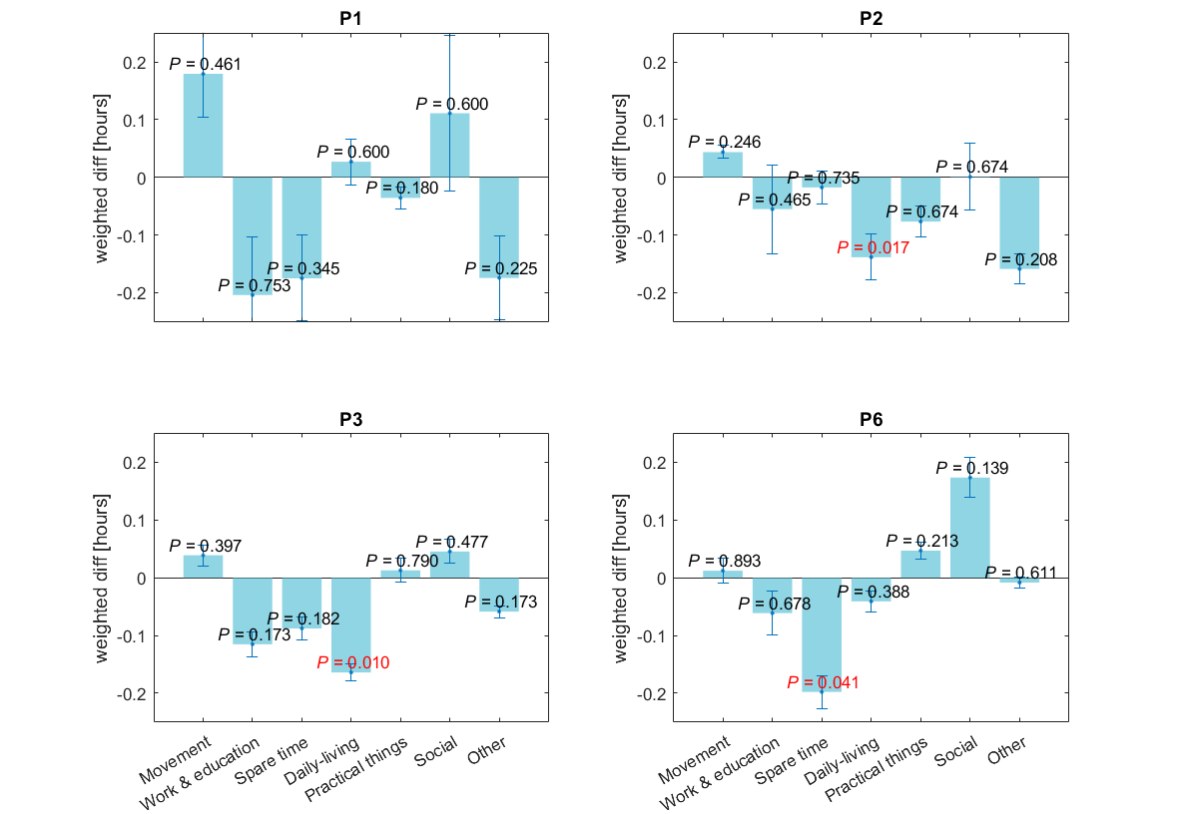
Four subplots, for each patient, illustrates the difference in the weighted hours between “good days” and “worse days” for each category. The error bar shows the SD. The result of a non-parametric Wilcoxon Signed-Rank on the weighted hour difference is visualized as an attached P value, colored red for the statistically significant results.

Time series of the daily average PS is shown in Figure 3. The median for each patient was 4.94, 5.67, 5.50, and 4.31 respectively, and is plotted as a dotted horizontal line. The *good days* group created by days above the median was for P1 Wednesday and Friday in week one and Tuesday until Friday week 2, with the rest classified as *worse days*. The largest *worse days* weight was on the last day and the largest *good days* weight on Thursday week two. The result, when comparing the 2 classes with the nonparametric Wilcoxon Signed-Rank, is visualized in Figure 4. The null hypothesis was rejected in 3 cases. There was a statistical significant difference for *Daily living* in P2 (*z*=–2.381, *P*=.017), *Daily living* for P3 (*z*=–2.589, *P*=.01), and *Spare time* in P6 (*z*=–2.045, *P*=.041). The negative direction in all the cases indicates that the patients had more hours of the given category during *worse days*.

**Figure 5:**
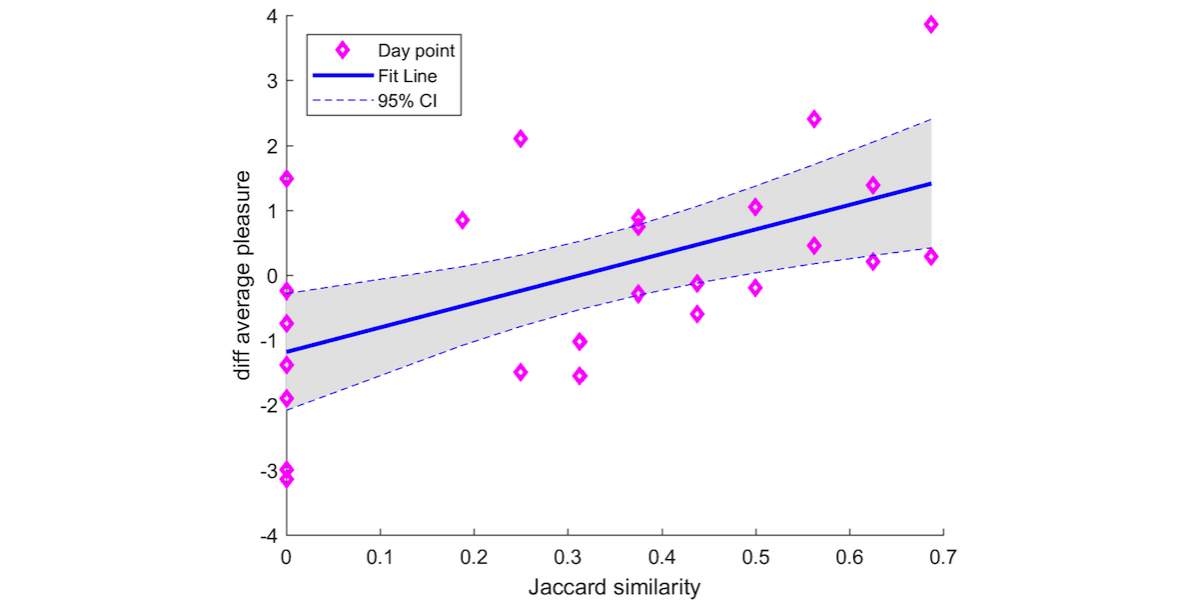
The change of the following day average pleasure score from the current day average pleasure score as a function of the Jaccardian similarity coefficient of the current day. The data is visualized as purple marks. The linear least square fit is shown as the blue line with its corresponding 95% CI as the dotted blue line.

The most frequent AC for each timeslot of each patient is shown in Multimedia Appendix 1. The JAC was calculated for each day, and the within-subject ANCOVA, testing the correlation value between JAC and the change in PS, was significant (correlation=.265, *P*=.029). A post hoc test of P3, visualized in Figure 5, was also significant (correlation=.317, *P*=.002). The zero point of the change was found to be JAC=.44, which indicates that the daily activities produced a positive change in PS the following day if the similarity between the day and the *diurnal rhythm* was >.44.

**Figure 6:**
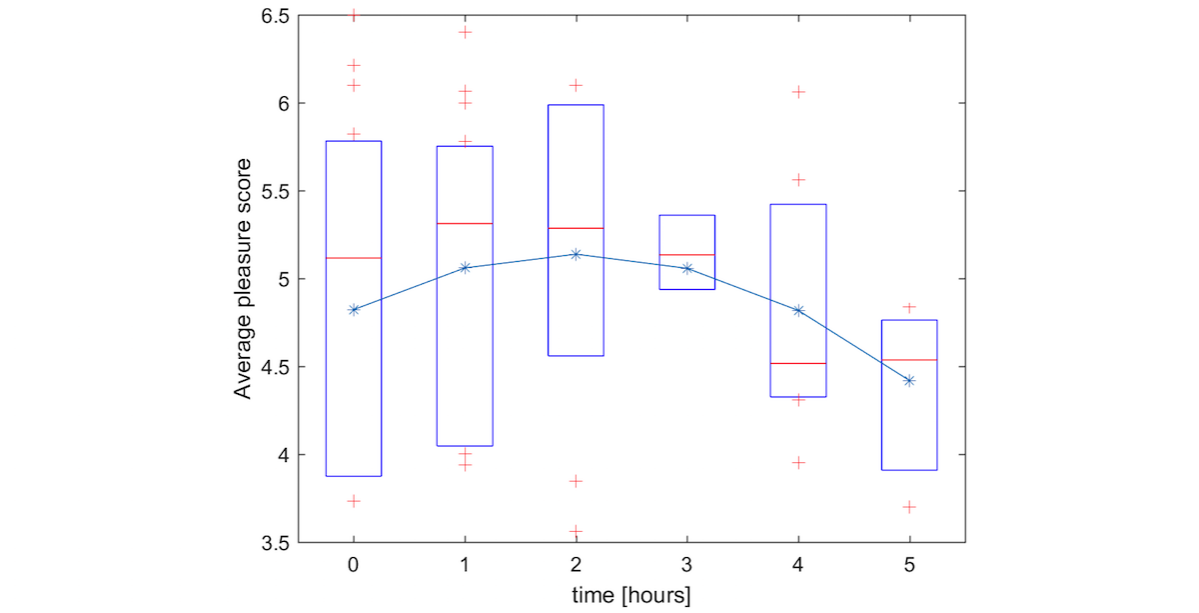
Across all subject, the days with 0 till 5 hours of “Social” activity is plotted with their corresponding average pleasure score. For each hour, a box-plot is visualized, which represents the median and the 25% and 75% quantiles. The blue line represents the fitted linear regression model.

A cross-sectional day analysis was performed where we looked at the number of hours of an AC and the corresponding average PS. A linear regression analysis revealed no significant parameters besides the intercept term, which represents the average PS at zero hours of the AC. However, a pattern was revealed for the linear regression model with second order parameter in the category *Social* (β_2_=–0.08, *t* (63)=–1.22, *P*=.23) and visualized in Figure 6 for further discussion.

**Table 1:** An overview of the patients, identified by their patient identification (ID). The amount of days with handwritten activity registrations is shown as “Days” and the number of reported activities as “Activities”, followed by the “Hourly compliance rate.” The agreement of the translation between the 2 independent researchers was assessed with Cohen kappa. The last column identifies the cases where pleasure scores were reported by the patients.

Patient ID	Age	Gender	Days of activity reporting	Activities reported	Hourly compliance rate	kappa	Pleasure score
P1	30	Female	12	185	96%	.78	Yes
P2	44	Female	17	251	92%	.78	Yes
P3	27	Female	28	399	89%	.77	Yes
P4	44	Female	51	680	83%	.84	No
P5	21	Female	7	108	96%	.79	No
P6	27	Female	19	306	95%	.87	Yes
P7	29	Female	19	301	99%	.82^a^	No

^a^This patient was object for reassessment due to an initial value kappa=.66 below the threshold kappa=.75.

**Table 2:** The distribution of hours within each translated activity category for the patients.

Patient ID	Movement %	Work & education %	Spare time %	Daily living %	Practical %	Social %	Other %
P1	6.77	20.83	13.54	23.44	1.56	21.35	12.50
P2	3.25	16.26	7.32	27.64	19.11	15.85	10.57
P3	7.75	11.75	18.00	32.50	14.25	11.25	4.50
P4	2.85	9.27	18.40	20.97	11.98	33.52	3.00
P5	8.04	0	2.68	15.18	18.75	55.36	0
P6	5.35	20.40	19.40	26.76	8.70	15.72	3.68
P7	5.98	19.93	9.97	39.20	2.99	16.28	5.65

## Discussion

To the best of our knowledge, no previous study has analyzed hourly sampled activity data with the purpose of a data-driven method to learn from the patients’ behavior. This analysis is useful for two goals: First, this data-driven approach provides an insight into the behavioral patterns of patients, which might be useful by therapists in BA therapy sessions. Second, since obtaining high-resolution activity data is challenging, this study and its statistical analysis have implications for how technology can be used in the design of BA technologies.

### Implications for the Use of Behavioral Activation in Therapy Sessions

A data-driven approach to analyzing behavior can provide an insight into the relationship between activity and the disease progression for patients on an individual level and more generally. In particular, this analysis can help identify (1) what activities are done on *good* and *bad days*, (2) what is the relationship between having a regular activity pattern and symptoms, and (3) and if there is a relationship between the amount of an AC and PS.

#### What Activities Are Done on Good and Worse Days?

In Figure 3 it is apparent that the patients’ mental state changes. P3 had two weeks with a stable PS, but that changed the last two weeks dramatically with variations from 1.80 to 6.90. To analyze the fluctuations, we separate the days that fluctuated below and above the median PS into two groups. The difference in the amount of AC for those two groups is illustrated in Figure 4.

All patients have registered more hours of *Movement* and *Social* in *good days*. The *Movement* category had the highest average PS across all subjects and days when looking at Figure 2. This was seen when inspecting the activity plan. For instance, P3 had a general PS of 5.5 during the day but the time slots registered as “*horse riding* ” was always in the upper range of 7.8-10.0. P1 had an average PS from 7-8 pm of 5.4 but then went for a “*night run* ” from 8-11 pm with an average PS of 7.0. This association can be related to the correlations studies on depressive symptoms and physical activity. In a study by Edwards et al [25], they found a significant increase in depression scores in the group of healthy young adults that were asked to minimize physical steps for one week. In a systematic review, Rohani et al [23] revealed a consistent negative correlation among studies between vigorous activity and depressive symptoms.

In the *worse day* s, the patients registered more hours within *Work & education*, *Spare time*, and *Other*. *Spare time* covered a majority of activities with low PS as visualized in Figure 2 and 4 such as P6: “*resting* ” and P3: “*home on the couch*.” These activities could represent sedentary depressed behavior unlike enjoyable activities that received high PS such as “*reading*,” and “*knitting*.” A greater, although nonsignificant, amount of *Work & education* –based activities during worse days, was a surprising observation. Usually, the likelihood of attending class or work is seen in days with less depressive symptoms [26]. Note, however, that a full-time work week in Denmark is 37 hours [27], and since the activity schema covers 112 hours including weekends, registration of work activity should correspond to 36/112 (32%). As shown in Table 2, no patients achieve this and only 4/153 (2.6%) days of activity data had more than 7 work hours. Hence, the considerable amount of work hours does not represent that the patient is back in a full-time work period.

In 3 cases there was a statistically significant difference in the number of hours between *good days* and *worse days*. Both P2 and P3 had a larger amount of *Daily living* AC during *worse days*. In the case of P3, when looking at the day with the largest *worse days* weighting with average PS=1.80, she had 2 hours of “*getting up* ” from the bed and eating “*breakfast*.” Alternatively, the Wednesday and Saturday of week 4 which were classified as *good days*, she merged the *Daily living* activities into a single hour slot: “*getting up+shower+breakfast*.” P2 had more hours of “*sleep* ” during the days with more weighting for *worse days*. For instance, Wednesday and Sunday of week two she had put in “*sleep* ” from 8 pm already. Moreover, when the patient reported lower PS, small and short activities tend to fill their day more such as P2 on Saturday week 2: “*home again* ” which on other days is registered as: “*cleaning–relaxing*.”

P6 had *Spare time* as a significant AC that occurs more on *worse days*. This was due to more registered hours of “*resting* ” contrary to *good days* that were filled with social activities such as watching TV with her son or doing practical work such as laundry.

Although the analysis was done on an individual basis and revealed distinct interpersonal behavior, some general AC patterns for all patients were revealed. For instance, the positive relationship between *Movement* and PS, and the negative relationship between *Spare time* and PS. The following investigation on *diurnal rhythm* was done as a within-subject analysis to continue the search for general relations.

#### Circadian Rhythm

We found that the more the patient followed their *diurnal rhythm* a better average PS was reported the following day, and the positive correlation was statistically significant. More precisely, scoring above a similarity of JAC=0.44 would yield a larger average PS the following day. This is in agreement with previous studies that use a comparable method. They find that keeping a regular location based circadian rhythm was significantly negatively correlated with depressive symptoms [28] and have a positive effect on mood [29]. The strong correlation from P3 mostly dominates the within-subject analysis. Interestingly, this is the patient with the most data entries (ie, 28 days) of the ones that provided PS scores as noted in Table 1.

#### Activity Category Frequency

A compelling observation was done by McKercher et al [30]. They show that male participants with depression were taking 7500-9999 steps per day, contrary to healthy controls that were in the lower or upper levels of respectively <7500 or >9999 step per day. Within personality traits, it is observed that introvert people have a lower threshold for outside stimuli such as social gatherings. After a certain number of hours, they would retreat [31]. Both observations demonstrate that there could exist an amount of, whether it is hours or steps, optimal for healthy living. In the same sense, we were interested to know if there is a relationship between the number of hours that are dedicated to a specific AC and the resulting average PS.

The cross-sectional model for all the participants did not reveal any significant relationship between the hours of an activity and the corresponding average PS. The AC, as already seen in Figure 4, has been shown to be highly interpersonal, which contributes to the high variability as depicted by the large box plots for each hour in Figure 6. Incorporating a larger dataset, by having patient registering more weeks or including more patients, could help to mitigate the interpersonal variance. Nevertheless, an interesting pattern was revealed with the *Social* AC as shown in Figure 6. We see a peak at 2 hours of social activity, which indicates that there may exist an optimal number of hours of a specific AC.

### Implications for the Design of Behavioral Activation Technology

The benefit of BA for managing depressive disorders had led to the design of several BA technologies in the form of standalone mobile phone apps within mental health, which are publicly available [32-34]. From this study, we want to draw three design implications related to (1) compliance rate, (2) semiautomatic activity collection, and (3) active use of data analysis for increased disease insight.

#### Designing for a High Compliance Rate

The usefulness of the system, as well as the validity of the collected data, is highly dependent on the engagement of the user. Figure 3 illustrates the problem with missing data. Data from P2 is missing the entire Friday in week two. We can observe that the average PS dropped dramatically from 6.21 to 3.85, but we do not know what happened. It could be that some events took place during that Friday, or maybe it was Saturday morning that was the cause of the low PS.

A core question in these BA technologies is, therefore, to what degree users will do the detailed hour-by-hour planning and registration of activities and associated mood or pleasure scores. This study has shown that the compliance rate of the paper-based diaries is rather high 5/7 (71.4%) is above 90% (Table 1). Note, a nonclinical sample of students who had to report data 8 times daily on a mobile phone had an average compliance rate of 87% [35]. This is contrary to the sample in this study, in which patients were included with mild to moderate depressive symptoms. Combining this with the fact that compliance rate is reported to be higher in digital solutions as compared to paper-based diaries [36], this yields the possibility of designing for a highly reliable collection of activity and mood data.

It is, however, essential to design for a high compliance rate as this does not come automatically. Therefore, easy-to-use and efficient ways to input data are needed (eg, using simple predefined activities and categories). Moreover, personalization to the individual user can be obtained by letting the system learn from the users and both reuse and suggest activities specific to a user and what he or she often does.

#### Designing for Semiautomatic Activity Collection

A consistent and standardized method of inputting activity data is required to design data-driven technology. Across all activity diaries, we identified 12 ways of reporting sleep such as “*sleep*,” “*sleeping*,” “*half-asleep,* ” and “*went to bed*.” Therefore, designing and applying standardized activity categories will achieve a consistent data entry, and ease the activity entry workload for the patient.

This study has suggested 6 such activity categories (see Textbox 1), which can be used in the design of technologies for BA. A question is the degree of coverage of these categories. For example, are they broad enough to cover all possible activities, and at the same time narrow enough to avoid loss of information? As seen in Table 2, only a few activities ranging from 0%-12.50% were classified as *Other*, which indicates that a clear majority of the patients’ activities could be categorized in the 6 categories.

Entries of “*transport* ” accounted for 96/116 (82.7%) of the *Other* classifications, which might make it a candidate for a seventh category. However, transportation can be inferred from the mobile phone embedded sensors [37]. Similarly, the *Daily living* category covered both the activity of “*eating dinner* ” as well as “*sleeping*.” These are 2 very different activities, which suggest that “*sleeping* ” should maybe be separated into a distinct category. Again, however, several studies have provided objective ways to infer sleep by using the mobile phone's sensors or a wearable sensor [38,39], which can be utilized in automatic detection and logging of sleep.

Hence, activities can be collected semiautomatic. Both entered by the user using a set of predefined activity categories as well as automatically inferred from sensors in a mobile phone.

#### Designing for Disease Insight

In a qualitative study within self-tracking for mental wellness [40] the participants reported that they felt more confident in the information they shared with their doctor because it was highly detailed and data-driven. The present study suggests opportunities for data-driven learning from self-tracked activities that could equip the user and his or her therapist with knowledge about personal behavioral traits. This feature is missing in current systems.

By taking inspiration from the type of statistical analysis done in this paper, a semiautomatic BA system could help a patient and the therapist with a personalized insight into the linkage between activities and disease progression. For example, a simple correlation analysis between activity types and mood (as shown in Figure 2) or the relationship between activity types and good or bad days, might help the patient understand what activities impact mood and vice versa. More advanced insight from a longitudinal collection of activity data might help the patient understand his circadian rhythms as shown in Figure 5 and finding an optimal level of different activities as shown in Figure 6.

### Limitations

This study was based on 7 patients, of which only 4/7 (57%) had reported pleasure scores. Despite covering an extended period, a total of 2,480 hours of activity sampling, this is a limited number of patients, which limits the generalizability of the analysis. Moreover, since these patients were part therapy session with a psychologist who asked the patient to report her daily activities, this is highly motivating for patients and hence leads to the high compliance rates revealed in the study.

In the analysis of the activity category frequency, we had to combine the data to a group level analysis, even though the theory behind it advocates for an individualized model. However, it is still important to note that the group level analysis used data on an hourly basis, which were based on 2,480 samples.

When transcribing specific activities into categories, some information gets lost. For instance, “*eating dinner* ” during the evening would be classified as *Daily Living* which is the same category if the person was sleeping instead. Similarly, when the person is talking on the phone, it would be classified as *Social* which is the same classification if the person went out to a cafe with a friend or family member. Hence, the classification is rather coarse-grained.

### Conclusions

Several insights were gained by performing statistical analysis on paper-based activity diaries. First, patients undergoing BA therapy had a much lower *Work & education* load than the general population, and they had a significant portion of *Daily living* related activities such as “*sleep* ” and “*eating*.” Second, in the days that the patient felt better, they had registered more hours of *Movement*, and *Social* related activities. Oppositely, the patients registered more hours of *Work & education*, *Spare time* and undefined activities when they felt an overall lower pleasure. However, there were several individual differences in the relationship between activities and corresponding pleasure, which was statistically significant. Third, there was a statistically significant increase in the pleasure score the next day if they followed their daily routine. Fourth, we did not find any general relationship between the amount of an activity you should do during a day and the resulting average pleasure. So even though *Movement* related activities produce a high pleasure score, it did not indicate that you should do as many hours as possible within the same day.

Some of these findings would be hard to discover during face-to-face consultations with a patient. Therefore, we suggest that a data-driven approach to learning behavioral traits could help to assist the psychologist in BA therapy sessions.

The compliance rate was above 90% for 5/7 (71.4%) of the patients, which indicates that this kind of activity registration is realizable to ask patients to perform. Moreover, the 6 proposed activity categories proved to cover 2114/2230 (94.8%) of all reported activities. This provides positive evidence for the feasibility of designing a digital platform supporting behavioral activation. A mobile phone solution could be designed to support such highly compliant and accurate data collection by applying a semiautomatic data collection approach. This can then provide personalized disease insight to the patient by revealing the connection between activity and mood, as outlined in this paper.

## Appendix

Multimedia Appendix 1 The most frequent AC for each timeslot (*diurnal rhythm*) of each patient

## Abbreviations

AC: activity category
ANCOVA: analysis of covariance
BA: behavioral activation
BD: bipolar disorder
CBT: Cognitive Behavioral Therapy
CSV: comma-separated values
JAC: Jaccardian similarity coefficient
PS: pleasure score

## References

[1] Kessler R, Avenevoli S, Ries MK (2001). Mood disorders in children and adolescents: an epidemiologic perspective. Biol Psychiatry.

[2] Merikangas K, He J, Burstein M, Swanson S, Avenevoli S, Cui L, Benjet C, Georgiades K, Swendsen J (2010). Lifetime prevalence of mental disorders in U.S. adolescents: results from the National Comorbidity Survey Replication--Adolescent Supplement (NCS-A). J Am Acad Child Adolesc Psychiatry.

[3] Akincigil A, Olfson M, Walkup J, Siegel M, Kalay E, Amin S, Zurlo K, Crystal S (2011). Diagnosis and treatment of depression in older community-dwelling adults: 1992-2005. J Am Geriatr Soc.

[4] Depp CA, Jeste DV (2004). Bipolar disorder in older adults: a critical review. Bipolar Disord.

[5] Wang PS, Simon G, Kessler RC (2003). The economic burden of depression and the cost-effectiveness of treatment. Int J Methods Psychiatr Res.

[6] Ferrari A, Charlson F, Norman R, Patten S, Freedman G, Murray C, Vos T, Whiteford H (2013). Burden of depressive disorders by country, sex, age, and year: findings from the global burden of disease study 2010. PLoS Med.

[7] (2016). The National Institute of Mental Health.

[8] McMahon K, Herr NR, Zerubavel N, Hoertel N, Neacsiu AD (2016). Psychotherapeutic Treatment of Bipolar Depression. Psychiatr Clin North Am.

[9] Bagby RM, Quilty LC, Segal ZV, McBride CC, Kennedy SH, Costa PT (2008). Personality and differential treatment response in major depression: a randomized controlled trial comparing cognitive-behavioural therapy and pharmacotherapy. Can J Psychiatry.

[10] Vallarino M, Henry C, Etain B, Gehue LJ, Macneil C, Scott EM, Barbato A, Conus P, Hlastala SA, Fristad M, Miklowitz DJ, Scott J (2015). An evidence map of psychosocial interventions for the earliest stages of bipolar disorder. Lancet Psychiatry.

[11] Richards DA, Ekers D, McMillan D, Taylor RS, Byford S, Warren FC, Barrett B, Farrand PA, Gilbody S, Kuyken W, O'Mahen H, Watkins ER, Wright KA, Hollon SD, Reed N, Rhodes S, Fletcher E, Finning K (2016). Cost and Outcome of Behavioural Activation versus Cognitive Behavioural Therapy for Depression (COBRA): a randomised, controlled, non-inferiority trial. Lancet.

[12] Kohn R, Saxena S, Levav I, Saraceno B (2004). The treatment gap in mental health care. Bull World Health Organ.

[13] Dimidjian S, Hollon SD, Dobson KS, Schmaling KB, Kohlenberg RJ, Addis ME, Gallop R, McGlinchey JB, Markley DK, Gollan JK, Atkins DC, Dunner DL, Jacobson NS (2006). Randomized trial of behavioral activation, cognitive therapy, and antidepressant medication in the acute treatment of adults with major depression. J Consult Clin Psychol.

[14] Shen GHC, Alloy LB, Abramson LY, Sylvia LG (2008). Social rhythm regularity and the onset of affective episodes in bipolar spectrum individuals. Bipolar Disord.

[15] Rohani DA, Tuxen N, Kessing LV, Bardram JE (2017). Designing for hourly activity sampling in behavioral activation. Pervasive Computing Technologies for Healthcare.

[16] Bardram JE, Rohani DA, Tuxen N, Faurholt-Jepsen M, Kessing LV (2017). Supporting smartphone-based behavioral activation: a simulation study. UbiComp '17 - Pervasive and Ubiquitous Computing.

[17] Wahle F, Kowatsch T, Fleisch E, Rufer M, Weidt S (2016). Mobile Sensing and Support for People With Depression: A Pilot Trial in the Wild. JMIR Mhealth Uhealth.

[18] Bardram JE, Frost M, Szántó K, Faurholt-Jepsen M, Vinberg M, Kessing LV (2013). Designing mobile health technology for bipolar disorder: a field trial of the monarca system. CHI '13.

[19] Rabbi M, Aung MH, Zhang M, Choudhury T (2015). MyBehavior: automatic personalized health feedback from user behaviors and preferences using smartphones. Ubicomp '15.

[20] Mørch MM, Rosenberg NK (2005). Kognitiv terapi: modeller og metoder.

[21] Lejuez CW, Hopko DR, Hopko SD (2001). A brief behavioral activation treatment for depression. Treatment manual. Behav Modif.

[22] (2014). US Bureau of Labor Statistics.

[23] Rohani DA, Faurholt-Jepsen M, Kessing LV, Bardram JE (2018). Correlations between objective behavioral features collected from mobile and wearable devices, and mood symptoms in affective disorders: A systematic review. JMIR mHealth and uHealth.

[24] Saeb S, Lattie EG, Schueller SM, Kording KP, Mohr DC (2016). The relationship between mobile phone location sensor data and depressive symptom severity. PeerJ.

[25] Edwards M, Loprinzi P (2016). Effects of a Sedentary Behavior-Inducing Randomized Controlled Intervention on Depression and Mood Profile in Active Young Adults. Mayo Clin Proc.

[26] Stewart WF, Ricci JA, Chee E, Hahn SR, Morganstein D (2003). Cost of lost productive work time among US workers with depression. JAMA.

[27] Christensen B (2014). Arb og helbred i Danmark.

[28] Saeb S, Zhang M, Karr CJ, Schueller SM, Corden ME, Kording KP, Mohr DC (2015). Mobile Phone Sensor Correlates of Depressive Symptom Severity in Daily-Life Behavior: An Exploratory Study. J Med Internet Res.

[29] Wirz-Justice A (2008). Diurnal variation of depressive symptoms. Dialogues Clin Neurosci.

[30] McKercher CM, Schmidt MD, Sanderson KA, Patton GC, Dwyer T, Venn AJ (2009). Physical activity and depression in young adults. Am J Prev Med.

[31] Aron E (1996). The Highly Sensitive Person: How to Thrive when the World Overwhelms You.

[32] Donker T, Petrie K, Proudfoot J, Clarke J, Birch M, Christensen H (2013). Smartphones for smarter delivery of mental health programs: a systematic review. J Med Internet Res.

[33] Grist R, Porter J, Stallard P (2017). Mental Health Mobile Apps for Preadolescents and Adolescents: A Systematic Review. J Med Internet Res.

[34] Huguet A, Rao S, McGrath PJ, Wozney L, Wheaton M, Conrod J, Rozario S (2016). A Systematic Review of Cognitive Behavioral Therapy and Behavioral Activation Apps for Depression. PLoS One.

[35] Wang R, Chen F, Chen Z, Li T, Harari G, Tignor S, Zhou X, Ben-Zeev D, Campbell AT (2014). StudentLife: assessing mental health, academic performance and behavioral trends of college students using smartphones.

[36] Stone A, Shiffman Saul, Schwartz Joseph E, Broderick Joan E, Hufford Michael R (2002). Patient non-compliance with paper diaries. BMJ.

[37] Widhalm P, Nitsche P, Brändle N (2012). Transport Mode Detection with Realistic Smartphone Sensor Data. ICPR2012.

[38] Chen Z, Lin M, Chen F, Lane N, Cardone G, Wang R, Li T, Chen Y, Choudhury T, Cambell A (2013). Unobtrusive sleep monitoring using smartphones.

[39] Ben-Zeev D, Scherer E, Wang R, Xie H, Campbell A (2015). Next-generation psychiatric assessment: Using smartphone sensors to monitor behavior and mental health. Psychiatr Rehabil J.

[40] Kelley C, Lee B, Wilcox L (2017). Self-tracking for Mental Wellness.

