# Supplementary figures and images for: Data-Driven Learning in High-Resolution Activity Sampling From Patients With Bipolar Depression: Mixed-Methods Study
Source: JMIR Ment Health. 2018 Jun 28;5(2):e10122. doi: 10.2196/10122 (PMC6043733; doi:10.2196/10122)

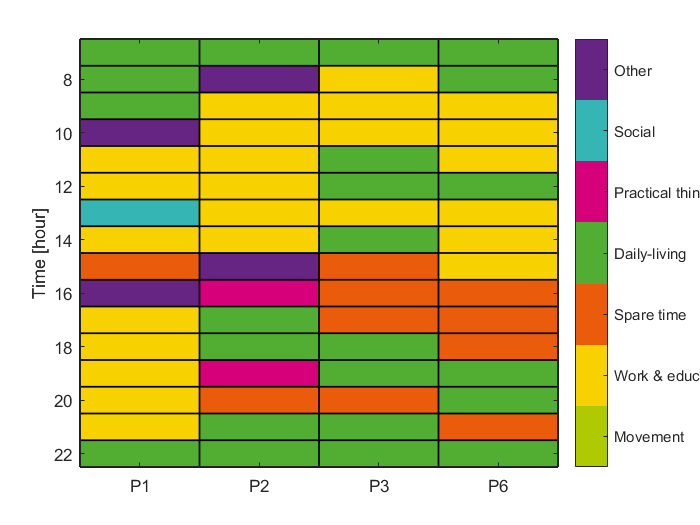

Supplement: Multimedia Appendix 1 [file mental_v5i2e10122_app1.png]
